# The correlation between microRNA490-3p and TGFα in endometrial carcinoma tumorigenesis and progression

**DOI:** 10.18632/oncotarget.7061

**Published:** 2016-01-28

**Authors:** Kai-Xuan Sun, Ying Chen, Shuo Chen, Bo-Liang Liu, Miao-Xiao Feng, Zhi-Hong Zong, Yang Zhao

**Affiliations:** ^1^ Department of Gynecology, The First Affiliated Hospital of China Medical University, Shenyang, P.R. China; ^2^ Department of Gynecology, The Fourth Affiliated Hospital of China Medical University, Shenyang, P.R. China; ^3^ Department of Biochemistry and Molecular Biology, College of Basic Medicine, China Medical University, Shenyang, P.R. China

**Keywords:** endometrial carcinoma, microRNA, TGFα, carcinogenesis, metastasis

## Abstract

MicroRNAs (miRNAs) are small non-coding RNAs that negatively regulate the translation of messenger RNAs by binding their 3′-untranslated region (3′ UTR). MiR-490-3p has been reported to be a suppressor in various human cancers; however, little is known about the biological functions of miR-490-3p in endometrial cancer (EC). In our study, we found that MiR-490-3p mRNA expression was significantly lower in ECs than in normal endometrial tissues. MiR-490-3p mRNA expression was also negatively associated with depth of invasion (mucosa *vs*. muscular and serosa) and lymph node metastasis (negative *vs*. positive) in EC. MiR-490-3p overexpression reduced proliferation; promoted G1 arrest and apoptosis; suppressed migration and invasion; and reduced TGFα, NF-kB, cyclin D1, survivin, matrix metalloproteinase 2 (MMP2) mRNA and protein expression, and improved Bax mRNA and protein expression. The dual-luciferase reporter assay indicated that miR-490-3p directly targeted TGFα by binding its 3′ untranslated region. MiR-490-3P transfection also suppressed tumor development and TGFα expression (as determined by immunohistochemistry and western blotting) in vivo in the xenograft mouse model. This is the first demonstration that miR-490-3P might act as a suppressor in EC tumorigenesis and progression by targeting TGFα. Our results provide a theoretical basis for the further study on the molecular target for endometrial cancer.

## INTRODUCTION

Endometrial carcinoma (EC) is one of the most common gynecological malignancies worldwide. Its incidence has recently increased worldwide, with a mortality rate of 2.4 per 100,000. In the USA, it is estimated that 52,630 women will be diagnosed with EC and 8,590 women will die from the disease in 2014 [1, 2]. Despite more than 70% cases being diagnosed at the early stage, as much as 28% of patients' have regional or distant metastasis. Unfortunately, their prognosis is usually poor, with a 5-year survival rate of < 40% [3]. Myometrial invasion and lymph node metastasis is the main cause of recurrence, and the causes of poor prognosis after surgery [4, 5]. However, to date, our knowledge about the tumorigenesis, invasion and metastasis of EC remains limited. Thus, It is important to identify new molecular mechanisms underlying the process of endometrial carcinogenesis and discover molecular targets.

Transforming Growth Factor-α (TGFα) is produced by macrophages, brain cells and epidermal cells, and can induce epithelial development [6, 7]. TGFα is upregulated in many tumors, and is associated with tumor invasion and metastasis, such as in hepatocellular [8], breast [9] and ovarian cancer [10]. In EC, TGFα is highly expressed in the tumor, and during invasion and metastasis [11–14], however, the mechanism regulating this high expression has not been determined.

MicroRNAs (miRNAs) are a class of single-stranded RNA, 22 nucleotide, non-coding, evolutionarily conserved RNAs that are able to bind to the 3′ untranslated region (UTR) of their target mRNAs, causing mRNA degradation or translational repression [15–19]. MiR-8 [20], miR152 [21], miR124 [22] and miR-376c [23] have been reported to regulate the expression of TGFα. Our software predicted that miR-490-3p could regulate the expression of TGFα via direct binding to its 3′ UTR. MiR-490-3P has been reported as a suppressor in various human cancers, including colorectal [24], gastric [25] lung [26] and bladder [27]; and as an oncogene in hepatocellular carcinoma [28]. However, little is known about the biological functions of miR-490-3p in EC. In this study we aimed to investigate whether miR-490-3p is a suppressor in human EC and to identify the direct target associated with TGFα.

## RESULTS

### The expression of MiR-490-3p in EC

**Figure 1:**
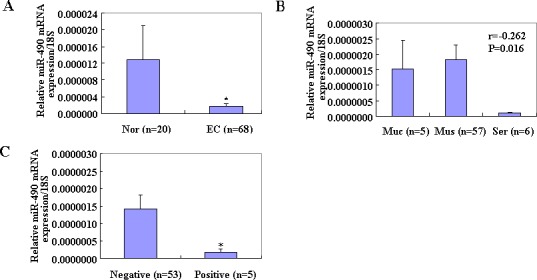
MiR 490-3p was decreased in tumor samples *P < 0.05 (A), as indicated by RT-PCR analysis Every sample was evaluated in triplicate and was negatively associated with depth of invasion (Muc *vs*. Mus and Ser, B) and lymph node metastasis (negative *vs*. positive, *P < 0.05, C) in EC. (Muc = mucosa; Mus = muscular; Ser = serosa).

To examine the expression levels of miR-490-3p in endometrial cancer, reverse transcription polymerase chain reaction (RT-PCR) analysis was performed among all the tissues samples. The expression of miR-490-3p was significantly downregulated in EC samples compared with normal samples (Figure 1A, *p* < 0.05), and was negatively associated with depth of invasion (mucosa *vs*. muscular and serosa, Figure 1B, *p* < 0.05) and lymph node metastasis (negative *vs*. positive, Figure 1C, *p* < 0.05) in EC.

### Effects of miR-490-3p transfection on EC cell phenotype *in vitro*

**Figure 2:**
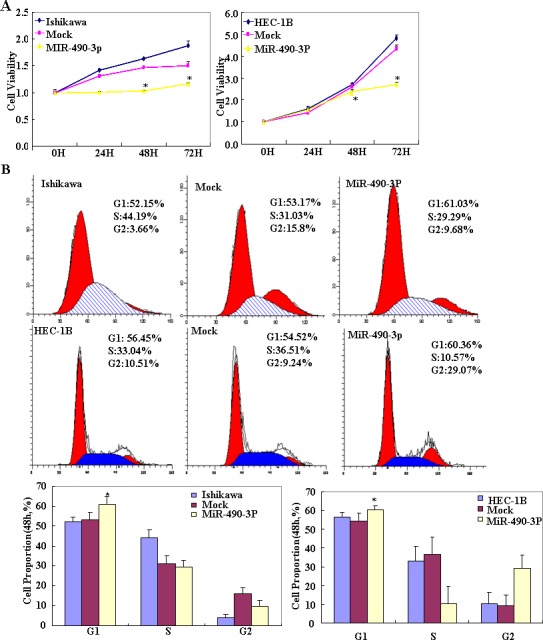
Following miR-490-3p transfection, HEC-1B, Ishikawa cell lines exhibited significantly slower growth (*P < 0.05, A), showed G1 arrest (*P < 0.05, B) Results are representative of three separate experiments; data are expressed as the mean ± standard deviation, *P < 0.05.

**Figure 3:**
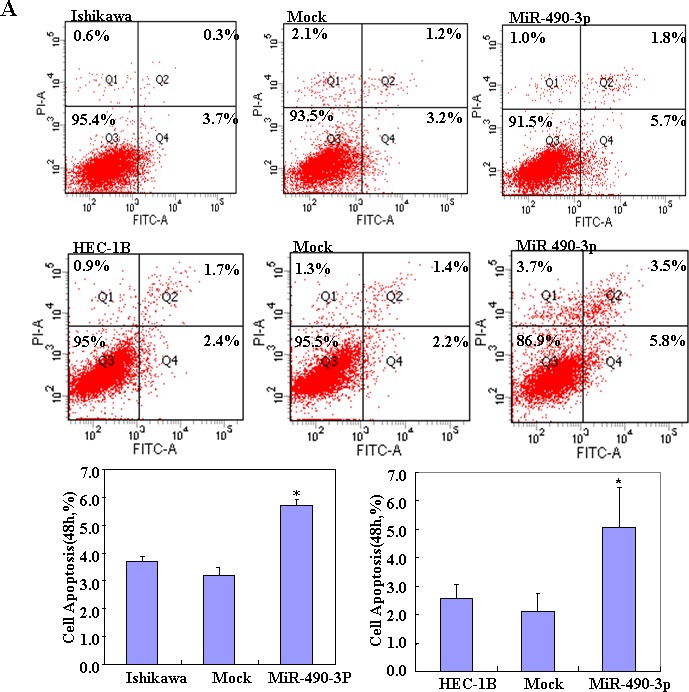
After transfection with the miR-490-3p mimics, showed early apoptosis (*P < 0.05, A) compared with the control and mock-transfected cells Results are representative of three separate experiments; data are expressed as the mean ± standard deviation, *P < 0.05.

**Figure 4:**
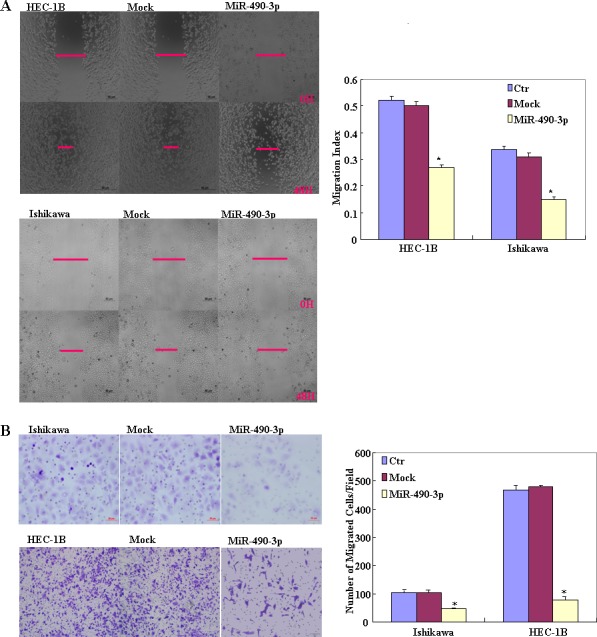
After transfection with the miR-490-3p mimics, showed lower migration in wound healing assays (*P < 0.05, A), and slower invasion in matrigel transwell assays (*P < 0.05, B), compared with the control and mock-transfected cells Results are representative of three separate experiments; data are expressed as the mean ± standard deviation, *P < 0.05.

To determine the effect of miR-490-3p overexpression in EC, we transfected endometrial cancer cell lines HEC-1B and Ishikawa with miR-490-3p-mimics. The expression of miR-490-3p increased after transfection and the ectopic expression of miR-490-3p inhibited cell proliferation in an MTT assay. The viability of the transfected cells was repressed (Figure 2A). MiR-490-3p transfection induced G1 arrest, as assessed by propidium iodide (PI) staining and flow cytometry (Figure 2B) and induced apoptosis, as shown by Annexin V-fluorescein isothiocyanate (FITC) staining (Figure 3A) and. We performed wound healing and transwell assays to measure migration and invasion. Transfection of miR-490-3p-mimics decreased migration and invasion compared with the scrambled control (Mock; Figure 4A-4B).

### Effects of miR-490-3p transfection on EC cell genotype *in vitro*

**Figure 5:**
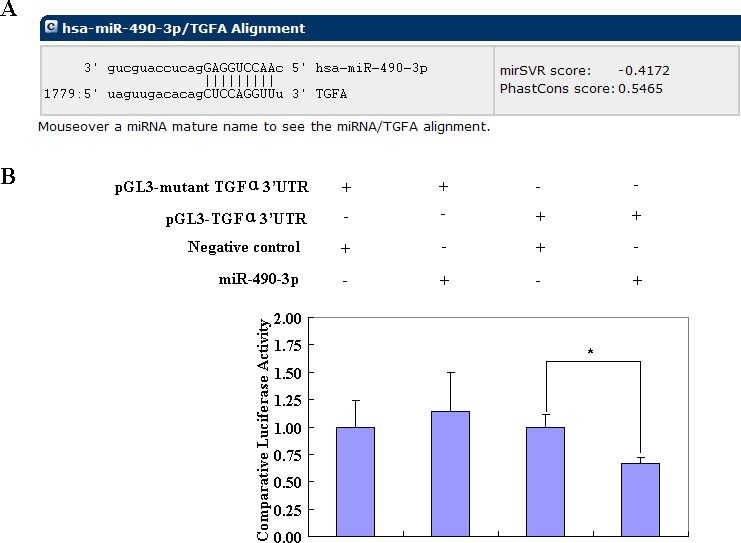
The bioinformatic software predicted that the 3′ UTR of *TGFα* was a direct target of miR-490-3p (A) A dual-luciferase reporter assay indicated that miR-490-3p directly targeted *TGFα* by binding its 3′ UTR (*P < 0.05, B).

**Figure 6:**
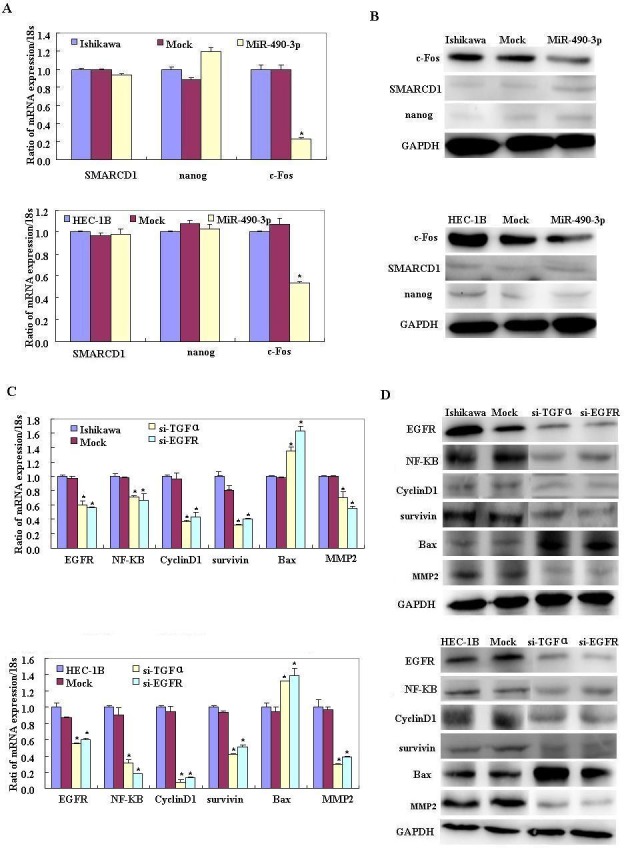

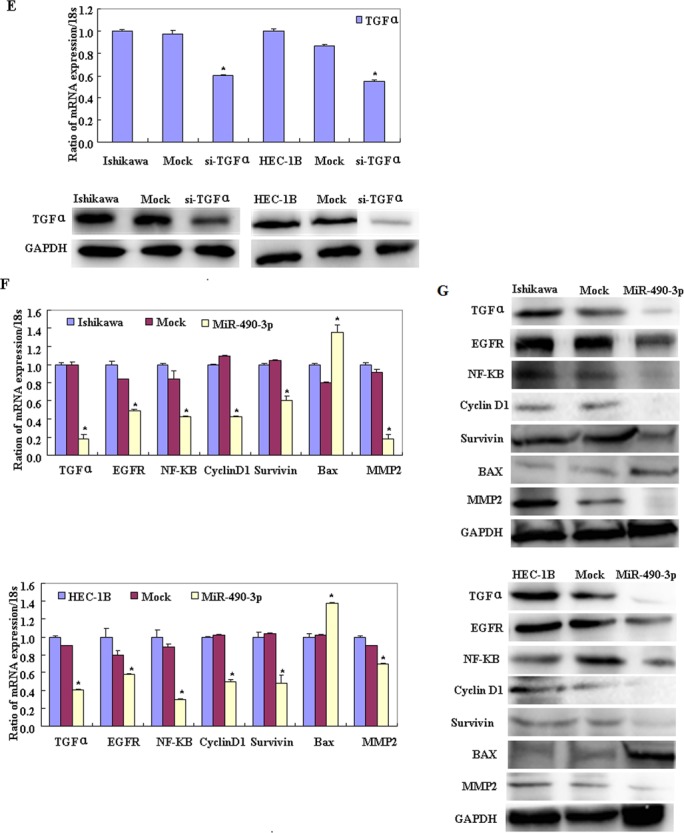
Reverse transcription (RT)-PCR and western blotting showed that miR-490-3p overexpression c-Fos decreased and nanog and SMARCD1 showed no significant differences (*P < 0.05, A-B). Then showed the change on TGFα, EGFR, NF-kB, Cyclin D1, Survivin, MMP2 and Bax in mRNA and protein level after miR-490-3p overexpression, si-TGFα and si-EGFR in EC cell lines.(C-G).

The bioinformatic software predicted that miR-490-3p has a direct target in the 3′ UTR of TGFα (Figure 5A). The dual-luciferase reporter assay indicated that miR-490-3p directly targeted TGFα by binding its 3′ UTR (Figure 5B). Reverse transcription (RT)-PCR and western blotting showed that miR-490-3p overexpression c-Fos decreased and nanog and SMARCD1 showed no significant differences. (Figure 6A-6B) Then showed the change on TGFα, EFGR, NF-kB, Cyclin D1, Survivin, MMP2 and Bax in mRNA and protein level after miR-490-3p overexpression, si-TGFα and si-EGFR in EC cell lines (Figure 6C-6G).

### MiR-490-3p inhibited tumor growth *in vivo*

**Figure 7:**
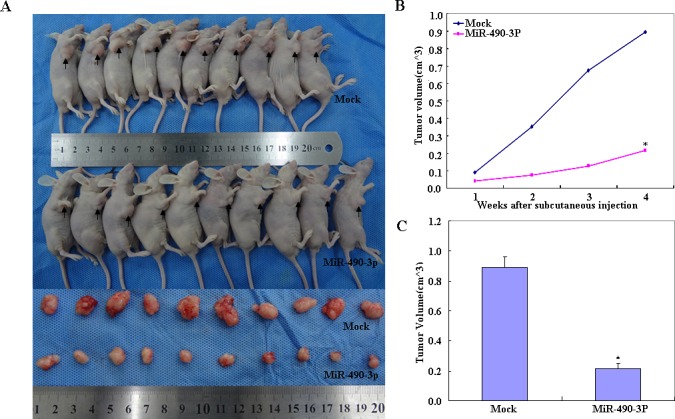
Compared with the mock control, nude mice treated with miR-490-3p showed a dramatic reduction in tumor size (A) and tumor xenograft growth from day 4 and week 2 onwards (*P < 0.05, B), while the deviation of tumor xenograft volume [DV] increased in the latter period (*P < 0.05, C)

Compared with the mock control, nude mice treated with hsa-miR-490-3p showed a dramatic reduction in tumor size (Figure 7A, *p* < 0.05) and tumor xenograft growth from day 4 and week 2 onwards (Figure 7B, day 4 p < 0.05; deviation of tumor xenograft volume [DV] *p* < 0.01, and week 2 *p* < 0.05; DV *p* < 0.01), while the DV increased in the latter period.

### MiR-490-3p downregulated TGFα expression in tumor xenografts *in vivo*

**Figure 8:**
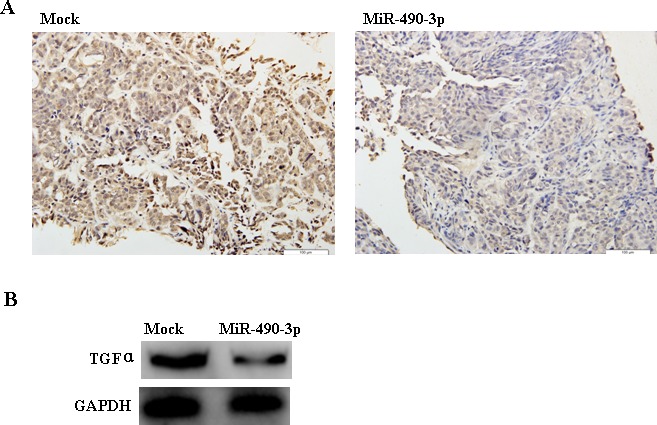
Immunohistochemistry (A) and Western Blotting (B) indicated that TGFα expression in the tumor xenografts of hsa-miR-490-3p-treated nude mice was decreased compared with that in mock-transfected nude mice

Immunohistochemistry (IHC) and Western Blotting indicated that TGFα expression in the tumor xenografts of hsa-miR-490-3p-treated nude mouse was decreased compared with that in mock nude mice (Figure 8A-8B).

## DISCUSSION

MiRNAs play important roles in carcinogenesis and have been the focus of much research in this area [30–32]. The targets of the miRNA have different activities, they are involved in many important cellular processes such as proliferation [33], apoptosis [34], differentiation [35, 36], metabolism [37], and tumor metastasis [38]. However, miRNAs have specific expression in different tissues, they had confirmed their potential use as diagnostic and prognostic markers [29, 30]. Many reports had showed the function of miR-490-3p in many cancers. Studies reported that miR-490 inhibits bladder cancer proliferation by targeting c-Fos [27], miR-490-3p could directly target nanog in mouse embryonic stem cell [29], and miR-490-3p exert suppressor in growth and metastasis on gastric cancer cells through directly targeting SMARCD1 [24]. However, miR-490-3p modulates cell growth and EMT in hepatocellular carcinoma cells by targeting ERGIC3 [28]. We found that miR-490-3p was expressed at a lower level in EC than in normal tissues and was negatively associated with depth of invasion and lymph node metastasis, after treated with miR-490-3p mimics, EC cells showed decreased cell proliferation, migration and invasion ability, increased the percentage of cells in G1 phase and decreased those in S phase and promoted the apoptosis of endometrial carcinoma cells. Besides, *in vivo* nude mice xenograft assays, the overexpression of miR-490-3p significantly inhibited tumor growth, these results all suggest that miR-490-3P may inhibit endometrial carcinoma tumorigenesis and progression. Those results consistent with miR-490-3p in colorectal [24], gastric [25], lung [26] and bladder [27] cancer, in contrast with hepatocellular carcinoma. At the same time, we detect the expression level of c-Fos, nanog and SMARCD1 in the endometrial carcinoma cells transfected with miR-490-3p through RT-PCR and Western blotting. The result demonstrated c-Fos decreased but nanog and SMARCD1 showed no significant differences. c-Fos was thought to enhance proliferation, motility and invasiveness of cancer cells. Yosuke konno etal reported that the mRNA expression of c-Fos levels were higher in EC than in normal endometrium, and showed that in aggressive EC cells, silencing of c-Fos by specific siRNAs can induce cell apoptosis and senescence, inhibit EMT-associated cell invasion, and reduce sphere formation ability [39]. So the anti-oncogene role of miR-490-3p in endometrial carcinoma may downregulating the expression of c-Fos. Besides, we found that miR-490-3P overexpression target TGFα in endometrial carcinoma. Our bioinformatics software prediction showed that miR-490-3p has a direct target in the 3′ UTR of TGFα, our dual luciferase reporter assays, the mRNA and protein expression levels of TGFα in miR-490-3p-transfected cells and in nude mice the tumor tissues of Hsa-490-3p group were decreased all verified this prediction.

TGFα is involved in cell growth and transformation of a class of cytokines, and exerts its biological function via the EGFRs family [40–41]. Increased expression of EGFR expression with TGFα found upregulated in pancreatic cancer, ovarian cancer, Wilms tumor and other tissues, both of which were positively correlated. Studies have suggested that in autocrine tumor cells, TFGα, acting on its own membrane receptors, stimulates the formation of its own value-added loop, which might play an important role in the process of tumor formation and development [42–44]. TGFα in pancreatic cancer cells regulates the proliferation and metastasis of cells through the MAPK pathway [30]. In breast cancer, TGFα signaling pathways are associated with related tumor cell invasion and the collagen matrix 9. In osteosarcoma the PI3K / Akt / NF-kB pathway was activated after TGFα treatment, which promoted osteosarcoma metastasis [45]. Previous studies observed that reduced TGFα induced own-regulation of MMP-2 and exhibited low invasive ability in prostate cancer [46]. TGFα also increases cell proliferation and avoids apoptosis in Ishikawa cells by upregulating cyclin D1 and reduced levels of TGFα inhibited cyclin D1, which led to cell cycle arrest G1or S and reduced amount of apoptosis; with corresponding increases in Bax and decreases in surviving [36, 47–51]. When we silenced TGFα and EGFR, the expression of NF-kB, MMP-2, Cyclin D1, survivin were all decreased in mRNA and protein level, while increased Bax expression. In short, TGFα is associated with proliferation, cell cycle apoptosis, invasion and metastasis in EC by NF-KB, MMP-2, Cyclin D1, survivin and Bax. After overexpressed of miR-490-3p in endometrial cancer cells the expression of TGFα was decreased, while EGFR and the downstream related genes including NF-kB, MMP-2, Cyclin D1, survivin were decreased in mRNA and protein level, while increased Bax expression. Above all, these results suggest that miR-490-3P may inhibit endometrial cancer tumorigenesis and progression through targeting TGFα.

In conclusion, miR-490-3p has a tumor suppressor role in endometrial cancer and with c-Fos and TGFα as direct target gene. Meanwhile, this study suggests that miR-490-3p could be considered as a potential target for the treatment and management of endometrial cancer in the future.

## MATERIALS AND METHODS

### Cell lines and culture conditions

The human EC cell lines HEC-1B and Ishikawa were purchased from the Tumor Cell Bank of the Chinese Academy of Medical Science (Peking, China). They were maintained in DMEM (HEC-1B), RPMI 1640 (Ishikawa) media with 10% fetal bovine serum (FBS), 100 U/mL penicillin and streptomycin in a humidified atmosphere of 5% CO2 at 37°C. The medium was changed every two or three days according to the recommended culture condition. All cells were harvested by centrifugation, rinsed with phosphate buffered saline (PBS), and subjected to total protein or RNA extraction.

### Transfection

Cells were cultured to 60-70% confluence and then resuspended in serum-free DMEM/RPMI 1640 at a concentration of 10^5^ cells/ml. Six-well plates were inoculated with 2 ml of cell suspension in each well and each group set three duplicate wells. MiR-490-3p mimics designed to mimic endogenous mature miR-490-3p (5′-CAA CCU GGA GGA CUC CAU GCU G-3′) were purchased from GenePharma (Shanghai, China), as well as scrambled oligonucleotides, which did not produce identifiable effects on miR-490-3p function, and were used as negative controls. Both were used as 5 nM diluted with 0.25 ml serum-free DMEM/RPMI 1640. Lipofectamine 2000 (Invitrogen, USA) transfection reagent (5μL) was diluted with 0.25 ml serum-free DMEM/RPMI 1640. Then, the diluted Lipofectamine 2000 transfection reagent was added to the diluted mimics, mixed gently and incubated for 20 min at room temperature. The cell suspension was changed with new medium and then added to the mixture of Lipofectamine 2000 and the mimics above, and incubated at 37°C and 5% CO_2_ for 6 h. Subsequently, the medium in each well was replaced with normal serum-containing medium and cultured for 48 h before to the following experiments.

### SiRNA treatment

TGFα and EGFR small interfering RNA (siRNA) transfect the EC cell lines which purchased from Sigma-Aldrich, USA. The target TGFα (5′-CCUUCCUACUUGGCCUGUdTdT-3′ and 5′-ACAGGCCAAGUAGGAAGGdTdT-3′) and EGFR (5′-GAGGAAAUAUGUACUACGAdTdT-3′ and 5′-UCGUAGUACAUAUUUCCUCdTdT-3′). Si-RNA was transfected into Ishikawa and HEC-1B cells using Lipofectamine 2000. Briefly, cells were seeded onto six-well plates (1×105 cells/well), and then transfected using 5μl siRNA mixed with 5 μl Lipofectamine in 1 ml medium without serum or antibiotics. The cells then 1 ml medium containing 20% serum was added to each well and were incubated for 48 h.

### Cell cycle analysis

The cells were trypsinized, collected at 10^6^ cells/ml, washed with PBS twice, and fixed in 70% ethanol at −20°C for 12 h. The cells were then washed with PBS twice and incubated with 3ul RNase (0.25 mg/mL) at 37°C for 1 h before being pelleted and resuspended in 50 μg/mL PI (KeyGen, China) 3ul and incubated at 4°C in the dark for 30 min. The PI signal was detected using flow cytometry.

### Apoptosis assay by flow cytometry

PI and fluorescein isothiocyanate (FITC)-labeled annexin V (KeyGen, China) were used to detect phosphatidyl serine externalization as an endpoint indicator of early apoptosis. Briefly, cells were washed with cold PBS, resuspended in binding buffer at 10^6^ cells/mL, and incubated with 5 μL annexin V-FITC and 5 μL PI. Samples were gently vortexed and incubated in the dark. Binding buffer was added to each tube for flow cytometry.

### Cell migration and invasion assay

Cell migration was measured using a wound-healing assay. Cells were seeded at 10^6^ cells/well in 6-well culture plates. After they had grown to confluence, the confluent monolayer in each well was scratched with a pipette tip, washed with PBS to clear debris, and cultured in FBS-free medium. The cells were then incubated in serum free medium for 48h. The individual gaps were observed and photographed using an inverted microscope at 0, 24, 48h at the same position of the wound; the wound area was measured using Image J software (National Institutes of Health, Bethesda, MD, USA). The wound healing rate = (Area of original wound - Area of actual wound at different times)/ Area of original wound × 100%.

Cell invasion assays were performed in 24-well, Matrigel-coated invasion chambers. In this assay, the upper champers were pre-coated with 30 μl Matrigel at a 1:10 dilution (BD Bioscience, San Jose, CA, USA), and incubated at 37°C for 4h. The EC cell lines were incubated post-transfection for 48 h, and then 5×10^4^ cells in 0.2 ml serum-free-DMEM/RPMI 1640 were added to the upper chambers (8-mm, Millipore), and 0.6 ml of 10% FBS-DMEM/RPMI 1640 was added as the chemoattractant to the lower chamber. The cells were incubated at 37°C for 48 h, after which, the non-invading cells were removed with cotton swabs. Cells that had invaded through the Matrigel and reached the bottom surface of the filters were fixed in methanol and stained with 0.1% crystal violet. The cells were counted in five random high-power fields at ×200 magnification per well. The experiment was performed in triplicate.

### Cell viability assay

Cell proliferation was analysed using a 3-(4,5-dimethylthiazol-2-yl)-2,5-diphenyltetrazolium bromide (MTT) assay. Cells were seeded into 96-well plates (5×10^3^ cells/well) directly and allowed to adhere. The medium changed to new medium with transfection reagent and incubated for 0, 24, 48, and 72h, respectively. After incubation with 20 ul of MTT (5 mg/ml, Sigma, USA) at 37°C for 4 h, the supernatants were removed, and 150 ul of dimethylsulfoxide (DMSO, Sigma, USA) was added to each well. The optical density (OD) of each well was measured at 490 nm. For each experimental condition, six wells were used, and the experiment was performed in triplicate.

### Tissue samples collection

Between January 2003 and April 2014, 68 endometrial adenocarcinomas (ECs), and 20 normal endometrial specimens from patients who underwent a hysterectomy to treat other benign diseases, were collected by surgical resection at The First Affiliated Hospital of China Medical University. All the EC patients were diagnosed following the criteria 58 underwent lymph node dissection of the International Federation of Obstetrics and Gynecology (FIGO 2009). The average age at surgery was 54 years (range 40-76 years). Informed consent was obtained from all subjects, and the China Medical University Ethics Committee approved the study.

### Reverse transcription polymerase chain reaction (RT-PCR)

Total RNA was extracted from endometrial carcinoma cell lines and endometrial cancer tissue using TRIzol (Takara, Japan). Total RNA (2 μg) was reverse transcribed to complementary DNA (cDNA) using avian myeloblastosis virus transcriptase and random primers (Takara, Shiga, Japan). The oligonucleotide primers for PCR were based on GenBank sequences. RT-PCR amplification of the cDNA was performed in 20 μL reactions according to the SYBR Premix Ex Taq™ II kit (Takara, Shiga, Japan); 18S rRNA was used as the internal control.

### Western blotting

Cells were harvested and lysed with ice-cold lysis buffer (Sigma, USA) and the protein concentration was determined using a protein assay kit (Bio-Rad Laboratories, Hercules, CA, USA). Denatured proteins (100 μg) were separated on 10% sodium dodecyl sulfate (SDS)-polyacrylamide gels, transferred to Hybond membranes (Amersham, Munich, Germany), and blocked overnight in 5% skimmed milk in Tris-buffered saline with Tween 20 (TBST). For immunoblotting, the membrane was incubated with antibodies against TGFα (1:200, Bioss), EGFR, NF-kB, Cyclin D1, Survivin, Bax, MMP2 and SMARCD1, nanog, c-Fos (Santa Cruz Biotechnology, Santa Cruz, CA, USA). The membranes were then rinsed with TBST and incubated with anti-mouse, or anti-rabbit IgG antibodies conjugated to horseradish peroxidase (1:5000; Dako, Carpinteria, CA, USA) for 2 hours. Bands were visualized on X-ray film (Fuji film, Tokyo, Japan) using Image Quant LAS 4000 (Fuji film, Tokyo, Japan) and ECL Plus detection reagents (Santa Cruz Biotechnology). GAPDH (Sigma-Aldrich) was used as a loading control.

### Immunohistochemistry

Consecutive tissue sections were deparaffinized with xylene, rehydrated with alcohol, and subjected to antigen retrieval by heating in target retrieval solution (Dako) for 15 min in a microwave oven (Oriental Rotor). The sections were quenched with 3% hydrogen peroxide for 20 min to block endogenous peroxidase activity. Non-specific binding was prevented by adding 5% bovine serum albumin for 5 min. The sections were incubated at 4C overnight with anti-TGFα antibodies, and then incubated with HRP-conjugated anti-rabbit antibodies (Dako) for 2h. After each treatment, the slides were washed three times with TBST for 5 min, and the binding sites were visualized with 3, 3′-diaminobenzidine. After counterstaining with Mayer's hematoxylin, the sections were dehydrated, cleared and mounted. Negative controls were prepared by omitting the primary antibody.

### Tumorigenicity assays in nude mouse

The Ethics Committee for Animal Experimentation of Chinese medical University approved all the experimental protocols.10^7^ HEC-1B cells suspended in 100 ul of serum-free medium were injected into right armpit of 4-week-old female BALB/C athymic nude mice. A group of mice (*n* = 10) received HEC-1B cells stably transfected with hsa-miRNA-490-3p. The other group mouse received mock-transfected HEC-1B cells. The tumor volume was measured every 3 days and all mice were sacrificed on day 28. All animal manipulations were performed in accordance with the National Institutes of Health Guide for the Care and Use of Laboratory Animals, and were approved by the China Medical University Animal Care and Use Committee.

### Dual-luciferase report assay

HEK293T Cells were grown to approximately 60% confluence in 24-well plates and co-transfected with TGFα 3′-UTR containing the putative miR-490-3p binding site or a mutant sequence designed based on the human TGFα mRNA sequence in GenBank and inserted into the downstream region of the firefly luciferase reporter (Promega, Madison, WI, USA). After 48 hours of incubation, luciferase activity was measured using the Dual-Luciferase Reporter System (Promega, USA), according to the manufacturer's instructions. The luciferase activities were normalized to that of Renilla luciferase. The results were expressed as the means ± SD of at least three independent experiments.

### Statistical analysis

Statistical evaluation was performed using the Spearman correlation test to analyze the rank data and the Mann-Whitney U test to differentiate the means of different groups. Cox's proportional hazards model was employed for multivariate analysis. A *p*-value of < 0.05 was considered statistically significant. SPSS 17.0 (SPSS, Chicago, IL, USA) software was employed to analyze all data.
